# Supplementary material for: Measuring Sedentary Behavior by Means of Muscular Activity and Accelerometry
Source: Sensors (Basel). 2018 Nov 17;18(11):4010. doi: 10.3390/s18114010 (PMC6263709; doi:10.3390/s18114010)
Supplement: Supplementary file 1 [file sensors-18-04010-s001.pdf]

sensors

Article

Measuring Sedentary Behavior by Means of Muscular Activity, Accelerometry and Artificial Intelligence

Roman P Kuster [1,2,*], Mirco Huber [3], Silas Hirschi [3], Walter Siegl [3], Daniel Baumgartner [2], Maria Hagströmer [1], and Wim Grooten [1]

[1] Division of Physiotherapy, Institute of Neurobiology, Care Science and Society, Karolinska Institutet, Stockholm, Sweden
[2] Institute of Mechanical Systems, School of Engineering, ZHAW Zurich University of Applied Sciences, Winterthur, Switzerland
[3] Institute of Energy Systems and Fluid Engineering, School of Engineering, ZHAW Zurich University of Applied Sciences, Winterthur, Switzerland
* Correspondence: roman.kuster@alumni.ethz.ch; Tel.: +41-58-934-6522

Received: 18 October 2018; Accepted: 15 November 2018; Published: 17 November 2018

Supplementary Materials

Table S1. List of the first 20 features for each developed decision tree model, including their holdout validity (AUC and MCC). The table reflects the stepwise feature inclusion for each model (e.g. AUC-6 sitting: the first 6 features listed in column "AUC model sitting" have an AUC = 0.85 and a MCC = 0.65). The last feature included in the models in Table 4 appear in bolt. Note that AUC model standing outperformed MCC model standing (not shown), and that one pre-selected signal feature (average signal power) was not used by any model within the first 20 features. The penalty used for each additional feature is not included in AUC and MCC values.

	AUC model sitting			MCC model sitting			AUC model standing		
#	Feature Names	AUC	MCC	Feature Names	AUC	MCC	Feature Names	AUC	MCC
1	CH2l_PowerAbove05Hz	0.61	0.21	X_autocor1sec	0.60	0.22	CH2r_peaksumrel	0.73	0.50
2	Z_peaksumrel	0.69	0.32	CH2l_PowerAbove5Hz	0.64	0.27	CH1l_PowerAbove05Hz	0.77	0.56
3	CH1r_PowerAbove05Hz	0.77	0.47	CH4l_harmpow	0.70	0.40	CH1r_harmpow	0.76	0.50
4	CH3l_midcrossingmedian	0.79	0.50	CH3l_harmpow	0.75	0.50	CH2r_midcrossingnumb	0.78	0.55
5	CH1r_peaknumb	0.81	0.52	CH4r_peaknumb	0.75	0.55	**CH2r_midcrossingmedian**	**0.80**	**0.59**
6	**CH3l_harmpow**	**0.85**	**0.65**	Z_peaksumrel	0.81	0.59	CH3r_peaksumrel	0.80	0.66
7	CH4r_std	0.85	0.64	CH4l_PowerAbove5Hz	0.81	0.63	X_PowerAbove5Hz	0.82	0.70
8	Y_midcrossingnumb	0.85	0.64	CH3r_midcrossingnumb	0.82	0.64	**X_peaknumb**	**0.84**	**0.71**
9	CH2l_PowerAbove5Hz	0.86	0.53	X_harmpow	0.84	0.65	CH3r_std	0.84	0.71
10	CH4r_harmfreq	0.90	0.59	**Y_PowerAbove5Hz**	**0.86**	**0.70**	CH1l_std	0.84	0.71
11	**CH3r_peaksumrel**	**0.90**	**0.60**	CH2r_PowerAbove05Hz	0.86	0.70	Z_std	0.84	0.71
12	CH3r_PowerAbove5Hz	0.90	0.60	CH3l_PowerAbove05Hz	0.86	0.70	CH3r_meanfreq	0.84	0.71
13	CH2r_PowerAbove05Hz	0.90	0.60	CH3l_std	0.86	0.70	CH1r_PowerAbove05Hz	0.84	0.71
14	CH2r_midcrossingmedian	0.90	0.60	CH3l_PowerAbove5Hz	0.86	0.70	CH3r_PowerAbove05Hz	0.84	0.71
15	Y_midcrossingmedian	0.90	0.60	CH2r_autocor1sec	0.86	0.70	Y_harmfreq	0.84	0.68
16	CH3r_harmfreq	0.90	0.59	CH3r_autocor1sec	0.86	0.70	X_midcrossingnumb	0.84	0.70
17	CH1l_PowerAbove5Hz	0.90	0.59	CH4r_autocor1sec	0.86	0.70	X_midcrossingmedian	0.84	0.71
18	CH1l_peaknumb	0.89	0.58	CH1l_autocor1sec	0.86	0.70	Y_PowerAbove05Hz	0.84	0.71
19	CH3r_PowerAbove05Hz	0.90	0.59	CH1l_midcrossingnumb	0.86	0.70	CH3r_PowerAbove5Hz	0.84	0.71
20	CH4l_midcrossingnumb	0.90	0.59	CH2r_midcrossingmedian	0.86	0.70	CH1l_PowerAbove5Hz	0.84	0.71

Abbreviations: AUC (Area under the ROC curve), MCC (Matthews correlation coefficient)

Channel Names: CH1r/l (EMG Channel 1 on right/left body side, forearm), CH2 (upper arm), CH3 (shoulder), CH4 (lower back)

Signal Features: std (Standard deviation), autocor1sec (one second lag-autocorrelation), peaknumb (number of prominent peaks), peaksumrel (sum of prominent peaks in relation to the median), midcrossingnumb (number of median crossings), midcrossingmedian (median time between adjacent median crossings), meanfreq (mean frequency), harmfreq (1st harmonic frequency), harmpower (power of 1st harmonic frequency), PowerAbove05Hz (power above 0.5Hz), PowerAbove5Hz (power above 5Hz).

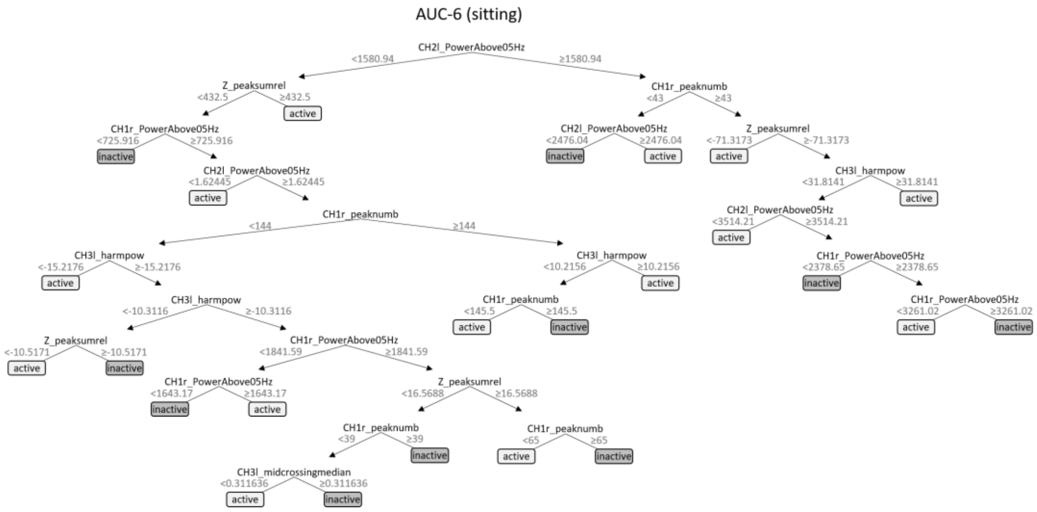

Figure S1. Final decision tree to separate sedentary behavior (inactive) from active sitting (active) using 6 signal features from 3 EMG channels (CH1r, CH2l, CH3l) and the thigh mounted accelerometer. The tree was developed using the Area under ROC Curve (AUC) as optimization criteria, and applied on all minutes correctly identified as sitting by the posture classification (figure 2). Tree performance is shown in Table 4. Abbreviations of channel names and signal features are listed in Table S1 footer.

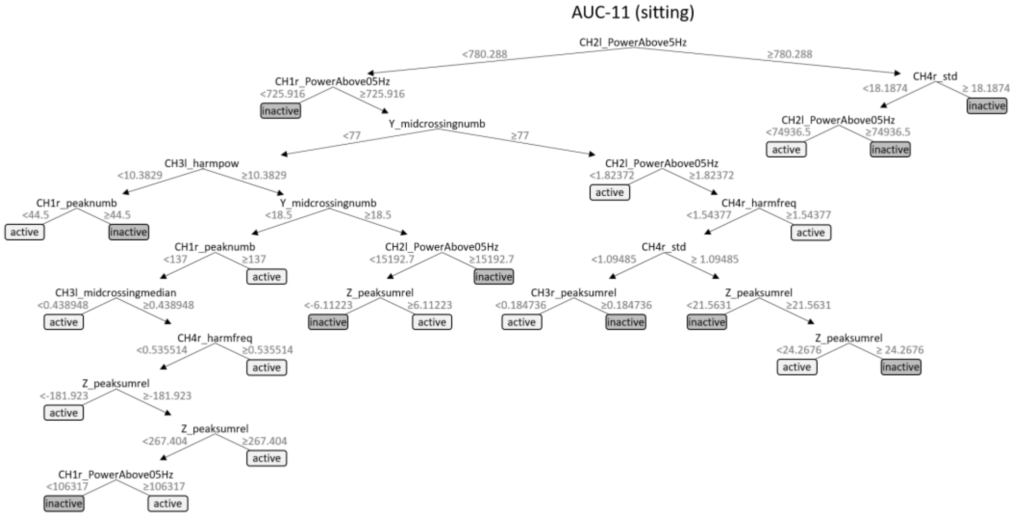

Figure S2. Final decision tree to separate sedentary behavior (inactive) from active sitting (active) using 11 signal features from 5 EMG channels (CH1r, CH2l, CH3r&l, CH4r) and the thigh mounted accelerometer. The tree was developed using Area under ROC Curve (AUC) as optimization criteria, and applied on all minutes correctly identified as sitting by the posture classification (figure 2). Tree performance is shown in Table 4. Abbreviations of channel names and signal features are listed in Table S1 footer.

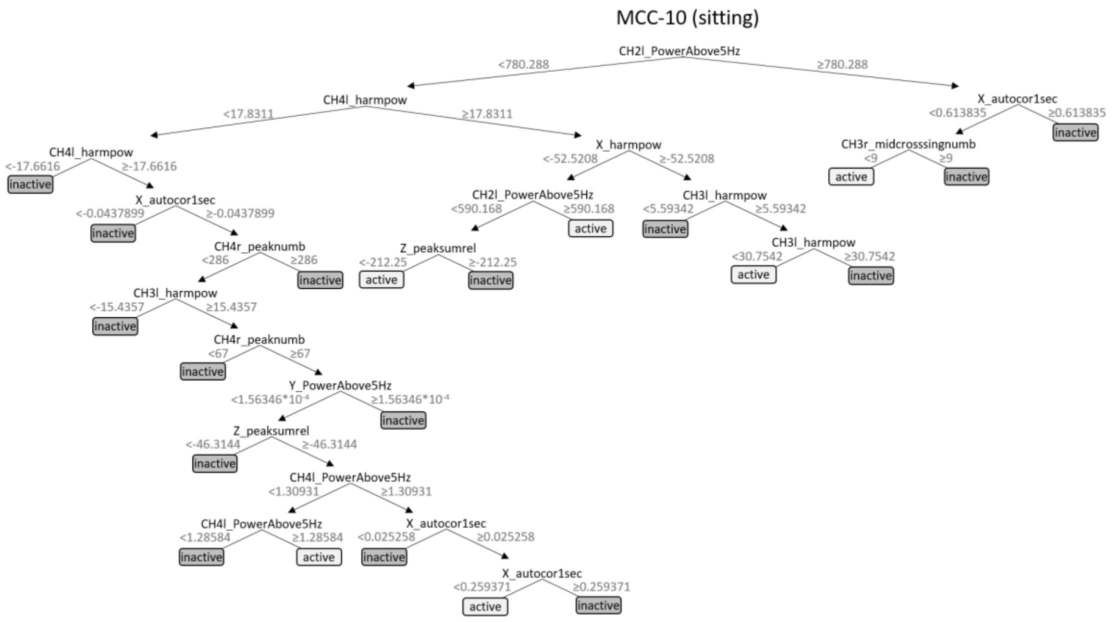

Figure S3. Final decision tree to separate sedentary behavior (inactive) from active sitting (active) using 10 signal features from 5 EMG channels (CH2l, CH3r&l, CH4r&l) and the thigh mounted accelerometer. The tree was developed using Matthews Correlation Coefficient (MCC) as optimization criteria, and applied on all minutes correctly identified as sitting by the posture classification (figure 2). Tree performance is shown in Table 4. Abbreviations of channel names and signal features are listed in Table S1 footer.

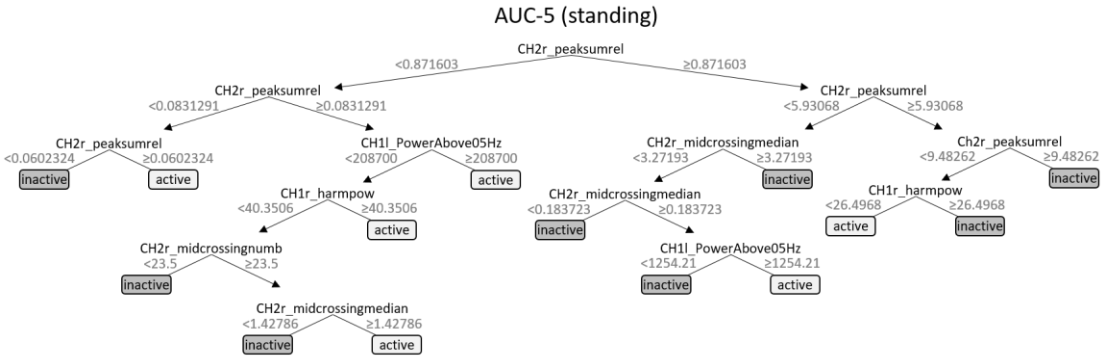

Figure S4. Final decision tree to separate inactive standing (inactive) from active standing (active) using 5 signal features from 3 EMG channels (CH1r&l, CH2r) and the thigh mounted accelerometer. The tree was developed using Area under ROC Curve (AUC) as optimization criteria, and applied on all minutes correctly identified as standing by the posture classification (figure 2). Tree performance is shown in Table 4. Abbreviations of channel names and signal features are listed in Table S1 footer.

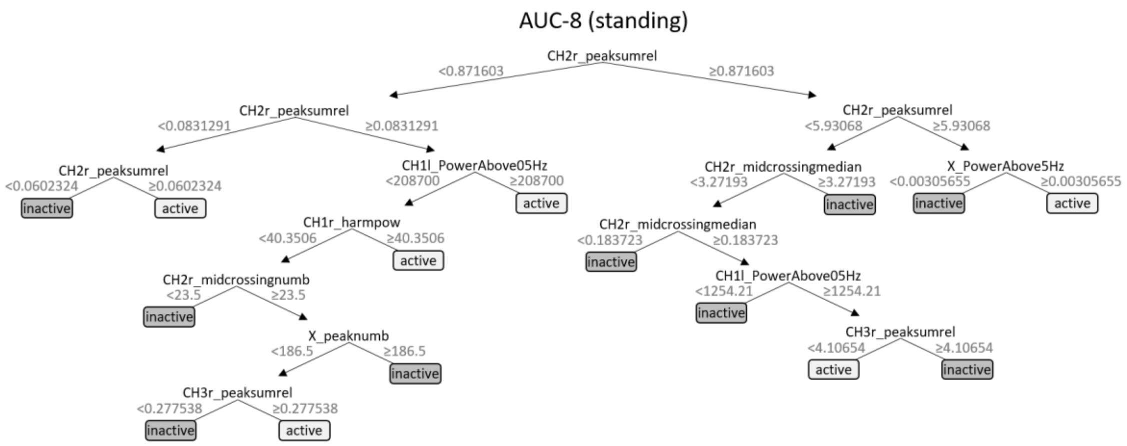

Figure S5. Final decision tree to separate inactive standing (inactive) from active standing (active) using 8 signal features from 4 EMG channels (CH1r&l, CH2r, CH3r&l) and the thigh mounted accelerometer. The tree was developed using Area under ROC Curve (AUC) as optimization criteria, and applied on all minutes correctly identified as standing by the posture classification (figure 2). Tree performance is shown in Table 4. Abbreviations of channel names and signal features are listed in Table S1 footer.
